# Epigenetics across the human lifespan

**DOI:** 10.3389/fcell.2014.00049

**Published:** 2014-09-09

**Authors:** Riya R. Kanherkar, Naina Bhatia-Dey, Antonei B. Csoka

**Affiliations:** Epigenetics Laboratory, Department of Anatomy, Howard UniversityWashington, DC, USA

**Keywords:** epigenetics, human lifespan, disease, environment, diet, development

## Abstract

Epigenetics has the potential to explain various biological phenomena that have heretofore defied complete explication. This review describes the various types of endogenous human developmental milestones such as birth, puberty, and menopause, as well as the diverse exogenous environmental factors that influence human health, in a chronological epigenetic context. We describe the entire course of human life from periconception to death and chronologically note all of the potential internal timepoints and external factors that influence the human epigenome. Ultimately, the environment presents these various factors to the individual that influence the epigenome, and the unique epigenetic and genetic profile of each individual also modulates the specific response to these factors. During the course of human life, we are exposed to an environment that abounds with a potent and dynamic milieu capable of triggering chemical changes that activate or silence genes. There is constant interaction between the external and internal environments that is required for normal development and health maintenance as well as for influencing disease load and resistance. For example, exposure to pharmaceutical and toxic chemicals, diet, stress, exercise, and other environmental factors are capable of eliciting positive or negative epigenetic modifications with lasting effects on development, metabolism and health. These can impact the body so profoundly as to permanently alter the epigenetic profile of an individual. We also present a comprehensive new hypothesis of how these diverse environmental factors cause both direct and indirect epigenetic changes and how this knowledge can ultimately be used to improve personalized medicine.

## Introduction

The literal meaning of the term epigenetic is “on top of or in addition to genetics.” The series of chemical tags that modify DNA and its associated structures constitute the epigenome, and include any genetic expression modifier independent of the DNA sequence of a gene. The genome defines the complete set of genetic information contained in the DNA, residing within the cells of each organism. The epigenome, on the other hand, comprises the complex modifications associated with genomic DNA, imparting a unique cellular and developmental identity.

The epigenome integrates the information encoded in the genome with all the molecular and chemical cues of cellular, extracellular, and environmental origin. Along with the genome, the epigenome instructs the unique gene expression program of each cell type to define its functional identity during development or disease (Rivera and Ren, 2013).

The epigenome also, in some sense, represents the ability of an organism to adapt and evolve through expression of a set of characteristics or phenotypes developed in response to environmental stimuli.

**Figure 1:**
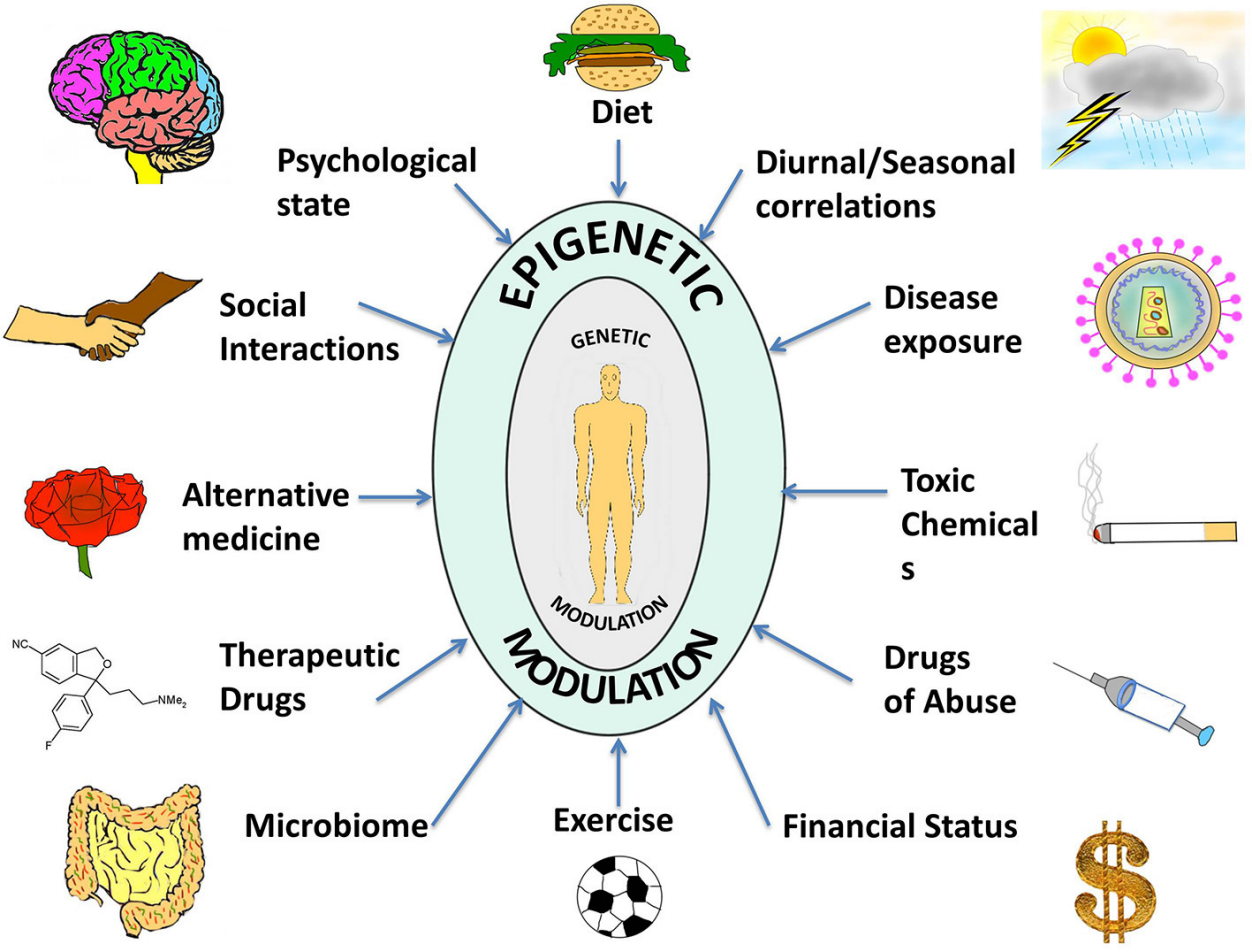
**A compilation of epigenetic influences on humans**. The figure represents a compilation of the various epigenetic influences on humans by different sources present in the environment. While some of these might be beneficial for health and behavior, others might be harmful and interfere with the body and mind creating an imbalance, which might manifest as a disease or psychological disorder. Some of the beneficial influences listed are exercise, microbiome (beneficial intestinal bacteria), and alternative medicine whereas harmful influences include exposure to toxic chemicals and drugs of abuse. Factors such as diet, seasonal changes, financial status, psychological state, social interactions, therapeutic drugs, and disease exposure might have beneficial or harmful effects depending on the specific nature of the influence. The environment thus complements and shapes human health. With the help of extended research in the field, we might be able to steer these influences in a positive way.

Thus, in contrast to the consistency of the genome, the epigenome is characterized by a dynamic and flexible response to intra- and extra-cellular stimuli, through cell-cell contact, by neighboring cells, by physiology, or entirely by the environment that the organism is exposed to Figure 1. Cytokines, growth factors, alterations in hormonal levels as well as release of stress-response and neurotropic factors are some examples of molecules that are modulated by the environment and which come under the category of epigenome modifiers. Ultimately, the environment presents these various factors to the individual that influence the epigenome, and the unique epigenetic and genetic profile of each individual also modulates the specific response to these factors (Figure 1).

Enzymatic activity in response to the environment promotes addition or removal of epigenetic tags on DNA and/or chromatin, sparking a cavalcade of changes that affect cellular memory transiently, permanently or with a heritable alteration. Some of the molecular mechanisms involved in this process are explained in more detail below.

## Mechanisms underlying epigenetics

As explained, every cell in the organism carries an identical genome, however, despite the stability of these instructions, the terminal phenotype within an organism is not fixed and deviation is caused by gene expression changes in response to environmental cues. DNA methylation, histone modification and RNA-associated silencing are the major ways these changes are controlled, which are described in more detail below.

### DNA methylation

The methylome is the genomic distribution of methylated DNA sequence present in a cell and is capable of undergoing modification with respect to the environment or the developmental stage. DNA methylation involves the covalent addition of a methyl group at position 5 of the pyrimidine ring of cytosine that is represented as 5-methyl C or C^Me^. Transcription of most protein coding genes in mammals is initiated at promoters rich in CG sequences, where cytosine is positioned next to a guanine nucleotide linked by a phosphate called a CpG site. Such short stretches of CpG-dense DNA are known as CpG islands. In the human genome 60–80% of 28 million CpG dinucleotides are methylated (Lister et al., 2009; Ziller et al., 2013). Chromatin structure adjacent to CpG island promoters facilitates transcription, while methylated CpG islands impart a tight compaction to chromatin that prevents onset of transcription and therefore, gene expression.

In CpG islands active C's are normally unmethylated and when an unmethylated cytosine spontaneously deaminates to uridine, it is converted back to cytosine by DNA repair mechanisms, thus preserving CpG sequences through evolution. The presence of 5-methyl C in a CpG island denotes an inactive promoter owing to the condensation of chromatin triggered by DNA methylation.

CpG sites are methylated by one of three enzymes called DNA methyltransferases (DNMTs). A variety of DNMTs are responsible for DNA methylation patterns established during embryogenesis. One type of DNA methyltransferase, DNMT1, is responsible for maintaining normal methylation patterns by copying them exactly between cell generations during replication. DNMT2 is associated with embryonic stem cells and potential RNA methylation. DNMT3a and DNMT3b are involved in *de novo* DNA methylation at CpG sites (Clouaire and Stancheva, 2008; Singh and Li, 2012).

### Histone modification

Histones are the core protein components of chromatin complexes, and they provide the structural backbone around which DNA wraps at regular intervals generating chromatin. The nucleosome represents the first level of chromatin organization and is composed of two of each of histones H2A, H2B, H3, and H4, assembled in an octameric core with DNA tightly wrapped around the octamer (Luger et al., 1997). Histones regulate DNA packaging with immense influence on the degree of chromatin compaction, influencing transcriptional activity as well as transcriptional silencing.

Histone modifications are post-translational changes on the histone tails, that are flexible stretches of N or C terminal residues extending from the globular histone octamer. Modifications of histones include acetylation of lysine residues, methylation of lysine and arginine residues, phosphorylation of serine and threonine residues, and ubiquitination of lysine residues present on histone tails, as well as sumoylation and ADP ribosylation. All of these changes influence DNA transcription. Addition or removal of methyl groups on DNA (see above) and histones and acetyl groups on histones are the prime mechanisms of changing the epigenetic landscape (Cedar and Bergman, 2009).

Histone acetylation is carried out by enzymes called histone acetyltransferases (HATs), that are responsible for adding acetyl groups to lysine residues on histone tails while histone deacetylases (HDACs) are those that remove acetyl groups from acetylated lysines. Generally, presence of acetylated lysine on histone tails leads to a relaxed chromatin state that promotes transcriptional activation of selected genes; in contrast, deacetylation of lysine residues leads to chromatin compaction and transcriptional inactivation.

### RNA silencing

RNA-associated silencing is a type of post-transcriptional gene modification during which the expression of one or more genes is downregulated or suppressed by small non-coding stretches of RNA, sometimes called microRNAs (miRNA) and small interfering RNAs (siRNA). Although microRNAs only represent 1% of the genome they have been estimated to target 30% of genes (Lewis et al., 2005). These RNAs can act as switches and modulators, exerting extensive influence within the cell and beyond. These RNAs fine-tune the gene expression as they act as specific modulators based on cell-type specificity of the organism during development as well as pathological conditions (Giraldez et al., 2005; Girardot et al., 2012; Baer et al., 2013). Also, miRNAs have been known to play a role in tumor suppression, apoptosis, cellular proliferation and cell movement which suggests that they can be manipulated in treating epigenetic diseases like cancer (Kala et al., 2013).

Putative mechanisms of RNA silencing include the ability of non-coding RNA to negatively regulate expression of target genes at the posttranscriptional level by binding to 3′-untranslated regions of target mRNAs resulting in their degradation (Singh et al., 2008).

All genes in every cell type are activated or silenced by an underlying interplay between these described epigenetic mechanisms. And as explained in the Introduction, exogenous epigenetic forces modify the endogenous inherited epigenetic pattern.

### Endogenous and exogenous epigenetic regulation of genes

**Figure 2:**
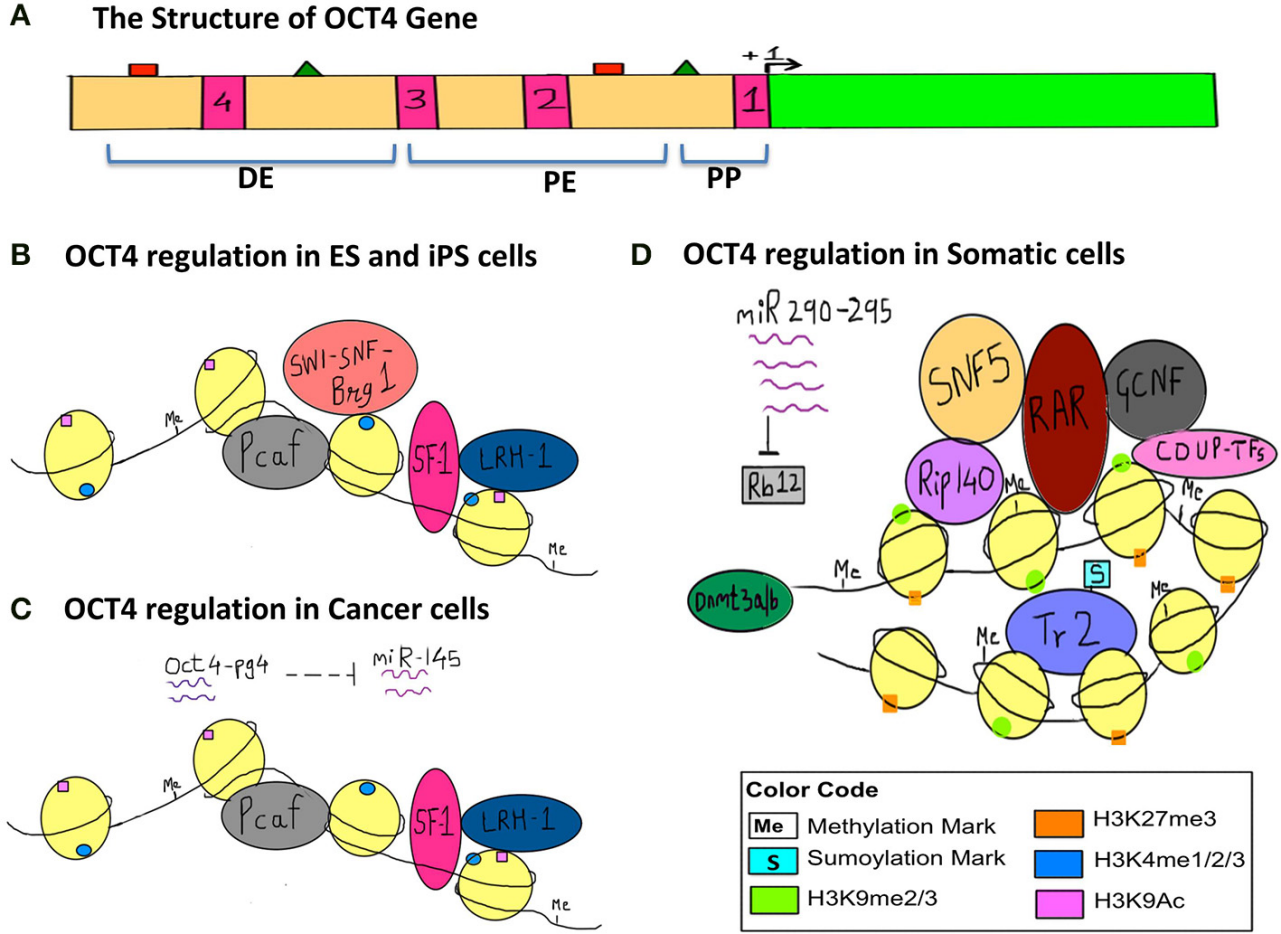
**Epigenetic regulation of OCT4 in stem cells, cancer cells and somatic cells**. The figure represents the various epigenetic mechanisms involved in regulating the gene expression of OCT4. **(A)** This section represents the structure of the OCT4 gene. The green bar represents the coding region. The beige bar represents the upstream region containing regulatory sequences. The four pink bars represent conserved regions; CR1 (−129 to −1), CR2 (−1510 to −1315), CR3 (−1956 to −1852), CR4 (−2557 to −2425) (data for human OCT4 gene). The green triangles represent the sites for binding of activators and the red squares represent the sites for binding of the repressors and these sites lie within the enhancers and promoter. +1 denotes transcription start site (TSS). (DE, Distal Enhancer −2057 to −1955; PE, Proximal Enhancer −1152 to −901; PP, Proximal Promoter −240 to +1) (This data is for the OCT4 gene from mouse ESCs since information for human OCT4 was insufficient). **(B)** This section represents the regulation of OCT4 in ESCs and iPSCs. OCT4 is highly expressed in these cells. Binding of transcriptional machinery induces OCT4 expression. This can be attributed to the hypomethylated promoter, enhancer region, which promotes binding of transcriptional activators and co-activators like Pcaf, LRH−1, SF−1, and Brg1 sub-unit of the SWI-SNF nucleosome-remodeling complex, which in turn facilitates the binding of RNA polymerase II. Activating histone marks like H3K4me and H3K9Ac are found on the histones surrounding the regulatory regions of the gene and they enhance the recruitment and binding of transcription factors thus inducing gene expression. **(C)** This section represents the regulation of the OCT4 gene in cancer cells, where similar to embryonic-like cells, it is highly expressed. In the case of hepatocellular carcinoma, an additional layer of epigenetic regulation is seen with the activity of the OCT4-pseudogene (pg)4, a non-coding RNA that protects the OCT4 transcript from inhibition by miRNA-145. Thus, OCT4-pg4 indirectly enhances the expression of OCT4 in cancer cells by acting as a protective shell for nascent OCT4 transcripts. The activity of the Brg1 sub-unit of the SWI-SNF complex is not necessary for OCT4 expression in cancer cells. **(D)** This section represents the regulation of OCT4 in somatic cells, which are differentiated and have a specialized function. Here OCT4 expression is repressed because of the hypermethylated promoter region and the repressive H3K27me and H3K9me marks. This results in compaction of the regulatory regions and facilitates the recruitment of other transcriptional repressors like RAR, GCNF, COUP-TFs, further compacting the regulatory regions, and making it inaccessible to the transcriptional machinery. SNF5, a core sub-unit of the BAF complex (SWI-SNF) acts as a switch between pluripotency and differentiation and promotes differentiation by binding to regulatory regions of OCT4. In addition, the sumyolation of orphan nuclear receptor Tr2 at Lys 238 results in its release from promyelocytic (PML) nuclear bodies. (In ESCs and iPSCs Tr2 is bound to PML nuclear bodies and acts as an activator). This in turn results in an exchange of co-repressor Rip-140 for co-activator Pcaf, making Tr2 a repressor. Also, miR-290 through miR-295 enhance methylation as they inhibit Rb12, which would otherwise inhibit the activity of *de novo* methyl transferase Dnmt 3a/3b.

In order to illustrate the endogenous epigenetic regulation of a gene, we will use the example of OCT4. OCT4 is the master pluripotency gene, which is regulated through different stages of human development, and its activation is necessary for maintaining pluripotency, whereas it must be silenced in order for a cell to differentiate (Kellner and Kikyo, 2010) (Figure 2). OCT4 is thus active in embryonic stem cells (ESCs), induced pluripotent stem cells (iPSCs) as well as in cancer cells, but is silenced in differentiated cell types. The three types of epigenetic modifications explained above i.e., DNA methylation, histone modification and RNA silencing are responsible for such regulation of OCT4 gene expression. This has been illustrated in detail along with the various epigenetic tags involved in its regulation in Figure 2.

In a similar way, let us consider the effect of exogenous epigenetic forces on the expression of OCT4. Vitrification, which is a commonly used method for cryopreservation, has been documented to alter the methylation patterns of the OCT4 gene. In two such cases, vitrification resulted in decreased methylation of the OCT4 promoter causing reduced gene expression in mouse blastocysts and the same was observed for mouse oocytes that underwent vitrfication followed by *in vitro* maturation (Milroy et al., 2011; Zhao et al., 2012). This explains how the external environment, in this case temperature, can lead to alteration of the epigenetic profile of one or many genes, ultimately causing differential gene expression.

### How do cellular biochemical changes cause epigenetic changes? a hypothetical mechanism

The effects of an epigenetic factor can be manifested as a global change in DNA methylation affecting multiple genes, or modified expression of very specific genes. The mechanisms and cellular pathways that are involved in the creation of these global or specific epigenetic changes are currently obscure. Below we describe a working hypothesis on how these changes might occur.

**Figure 3:**
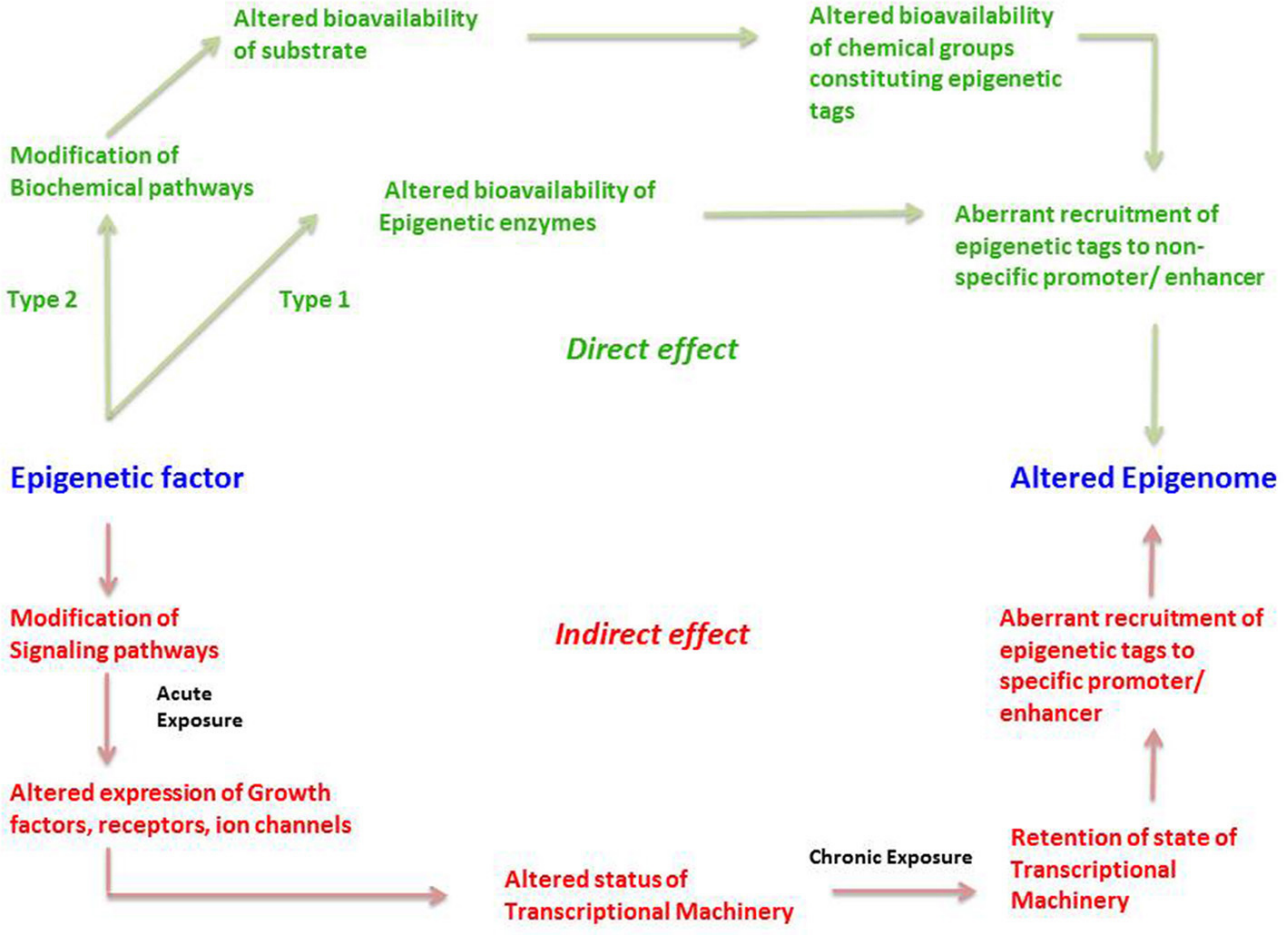
**The direct and indirect epigenetic pathway**. The figure represents two different routes through which an epigenetic factor can modify the epigenome leading to altered gene expression. Epigenetic effects exerted by an external factor or intrinsic environment can lead to direct and indirect effects on the epigenome. Green represents direct effects of an epigenetic factor and red represents indirect effects of an epigenetic factor. The direct pathway can operate in two different ways; namely Type1 and Type 2. In the Type 1 direct pathway, the epigenetic factor *directly* exerts an effect on the epigenetic enzymes such as DNMTs, HDACs, HATs, HMTs, HDMs, etc. such that there is an altered bioavailability of these enzymes in the cell. A Type 2 direct effect is when an epigenetic factor interferes with a biochemical pathway such that there is altered availability of a metabolite required for constituting an epigenetic tag. Both cases can result in aberrant or inadequate recruitment of epigenetic tags in a random fashion to non-specific promoters, ultimately establishing an altered epigenetic profile. In the indirect pathway, the epigenetic factor *indirectly* exerts an effect on the epigenome by first interfering with any signaling pathways of the cell. An acute exposure to the epigenetic factor can cause altered expression of growth factors, receptors, ion channels and so on resulting in non-homeostatic cellular processes. This in turn might lead to an altered status of transcriptional machinery (bound or unbound to the promoter/enhancer) and its bioavailability in a cell. A chronic exposure to the epigenetic factor might lead to retention of such state of transcriptional machinery (bound or unbound to the promoter/enhancer) causing altered gene expression as well as aberrant recruitment of epigenetic enzymes, leading to permanent addition or removal of epigenetic tags to specific promoter/enhancers. This consequently establishes an altered epigenetic profile.

We propose that an epigenetic factor can act through either a *direct* or an *indirect* mechanism (Figure 3). A direct effect could happen in two ways; which we term Type 1 and Type 2. Type 1 direct effect occurs when the epigenetic factor *directly* alters the state of epigenetic enzymes—either by binding to them and preventing them from carrying out their normal function, damaging them in some other way, or upregulating them. The altered bioavailability of epigenetic enzymes then results in aberrant recruitment of epigenetic tags to promoters and enhancers on a genome-wide scale. Such a direct effect would be effective across the entire genome, not affecting any specific gene but resulting in a randomly altered epigenome. An example of a directly acting epigenetic factor is the antihypertensive hydralazine, which inhibits DNA methylation.

Type 2 direct effects occur when an epigenetic factor causes a change in a biochemical process that results in an altered availability of a substrate, intermediate, by-product or any other metabolite participating in the biochemical pathway, that is used to make up epigenetic tags (for example acetate). This in turn leads to altered availability of epigenetic tags, in this case acetyl groups on histones, which leads non-specific modification of the epigenome (Figure 3).

**Figure 4:**
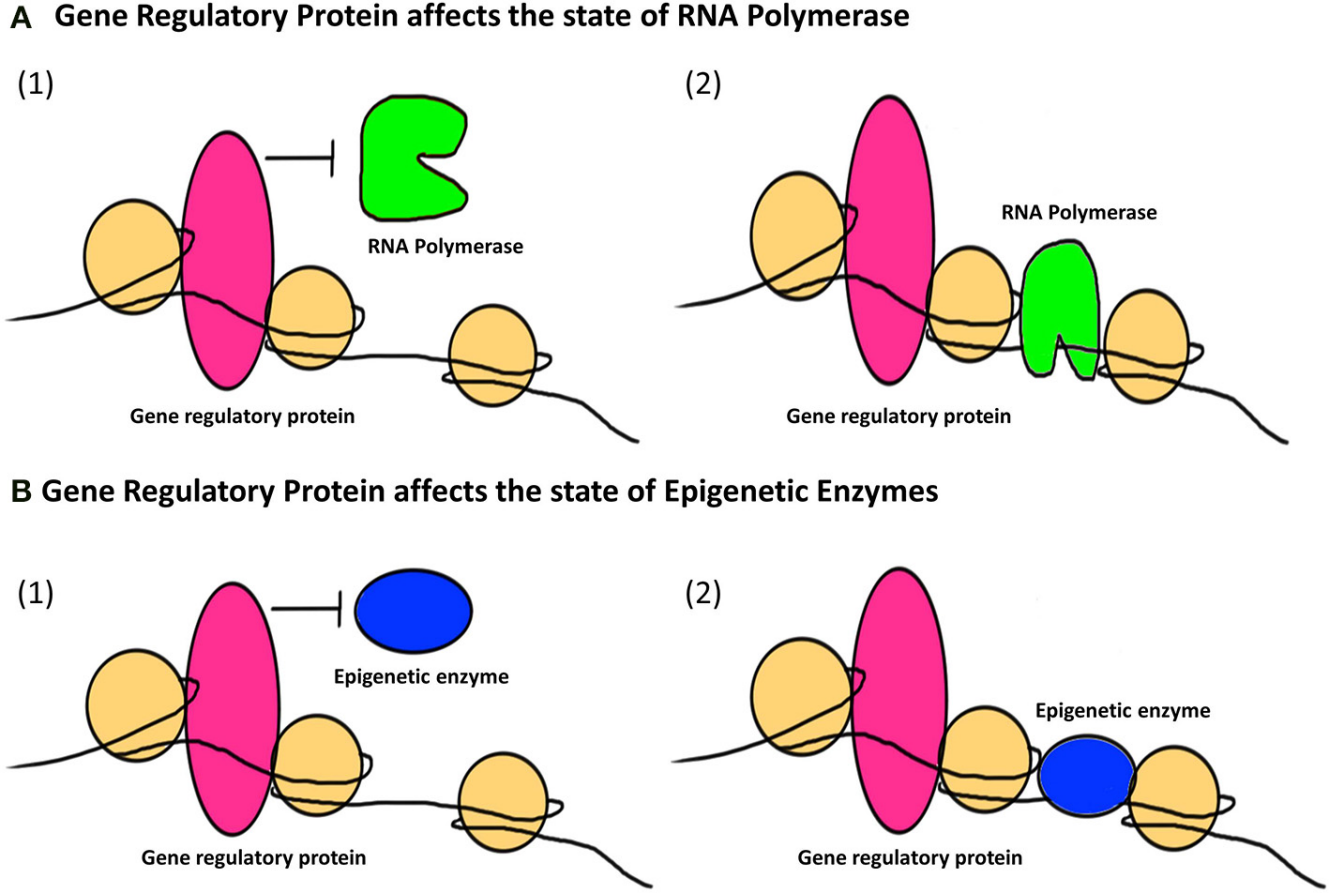
**The indirect epigenetic pathway**. An epigenetic factor operating through an indirect pathway interferes with transcriptional machinery. Chronic exposure to an epigenetic factor can lead to the retention of an already altered state of transcriptional machinery. The transcriptional machinery (bound or unbound to the gene regulatory regions i.e., promoters/enhancers) includes a number of proteins like transcription factors, activators and co-activators, repressors and co-repressors and nucleosome or chromatin remodeling complexes. For simplicity, these proteins are Fectively termed as “Gene regulatory protein” for this figure. **(A)** A gene regulatory protein might affect the status of RNA Polymerase (1) By inhibiting it from binding to a transcriptional apparatus or forming one or (2) by facilitating the binding of RNA Polymerase as well as formation of a transcriptional apparatus essential for the initiation of transcription. **(B)** A gene regulatory protein might also affect the status of epigenetic enzymes like DNMTs, HDACs, HATs, HMTs, HDMs, etc., which are responsible for the addition or removal of epigenetic tags (methyl group on DNA or histone, acetyl group on histones) on gene regulatory regions. This can be (1) By inhibiting epigenetic enzymes from binding to the gene regulatory regions and hence prohibiting the addition or removal of epigenetic tags (2) By facilitating the binding of epigenetic enzymes to gene regulatory regions and hence allowing the addition or removal of epigenetic tags. Such retention of a gene regulatory protein due to chronic exposure to an epigenetic factor might result in a permanent change in the epigenetic profile and/or gene expression of a specific gene affected by that epigenetic factor through an indirect pathway.

The second major way that factors can cause epigenetic changes is by what we term *indirect* mechanisms (Figures 3, 4). A biphasic mechanism is postulated for indirect effects in which acute exposure to a factor influences cellular signaling pathways that leads to altered expression of growth factors, receptors and ion channels, which in turn alter transcription factor activity at gene promoters. With more chronic exposure, the transcription factors and other gene regulatory proteins, in addition to altering gene expression activity, actually recruit or repel epigenetic enzymes to/from the associated chromatin, resulting in the addition or removal of epigenetic tags (Figure 4). In this way cells adapt to the persistent gene expression changes by causing permanent modifications to DNA methylation and chromatin structure, leading to enduring alteration of the affected epigenetic network (Figures 3, 4). An example of an indirectly acting factor is the drug isotretinoin, which has transcription factor activity.

Epigenetic factors known to cause such direct and indirect effects are well-documented but their exact mechanism has not been accurately elucidated. For example, nutritional interventions in adulthood like caloric restriction can induce epigenetic changes that have the potential to alleviate age-related diseases (Bacalini et al., 2014). In caloric restriction food intake is intentionally reduced by 30–50% (Zakhari, 2013) and has been shown to delay the aging process in mice by decreasing the levels of histone deacetylase 2 (HDAC2) which otherwise increases during the normal aging process (Chouliaras et al., 2013). Thus, this is an example of direct effect by an epigenetic influence (caloric restriction).

Another example is curcumin, a polyphenol found in turmeric, which can be regarded as a dietary epigenetic factor. It is an inhibitor of the Histone acetlytransferase p300/CBP (co-activator) and GATA4 (a zinc finger transcription factor), which leads to decrease in nuclear acetylation induced during myocardial cell hypertrophy (Morimoto et al., 2008). The possible mechanisms through which curcumin exerts such an effect might be through allosteric regulation of p300 or through interfering with nuclear signaling pathways like transcriptional activation by NF-κB (Morimoto et al., 2008). Hence the epigenetic effect of curcumin is an example of a combined direct and indirect effect.

## Epigenetics across the human lifespan

In this main section of the paper, we describe the major epigenetic milestones in the human lifespan, integrated chronologically with the various environmental factors that affect the human epigenome and the interaction with these milestones (**Figure 6**).

In differentiated cells, signals fine-tune cell functions through changes in gene expression across the lifespan. A flexible epigenome allows us to adjust to changes in the world around us, and to “learn” from our experiences. In many ways, epigenetic expression can be thought of as the “software” of the genome and directs embryogenesis and development, as well as influences the development of an individual's body and brain after birth. Unique sets of genes are induced or silenced epigenetically during different stages of life and these are responsible for the development and maturation of the individual through orchestrated events in combination with input from the environment. Any kind of epigenetic factors influencing genes or gene expression networks during life stages can result in an imbalance in the regulation process, and might have a life-long effect on the individual. While such flexibility gives rise to beneficial adaptability to environmental conditions, it likewise allows weaknesses to integrate and exert negative and diseased outcomes on both individual and evolutionary scales.

We have used data from human studies in most cases in this review, but in some cases where such data is sparse or unavailable, we have supported our explanations with data from studies on rodent and/or other animal models.

### From the periconceptional environment to birth

For most genes, total reprogramming is necessary very soon after conception in order to start with an epigenetic “clean slate,” which then allows all of the specialized cells derived from the egg and sperm to develop with stable cell-specific gene expression profiles and remain properly differentiated. This happens in the fertilized egg: global DNA demethylation is followed by remethylation to reprogram the maternal and paternal genomes for efficient gene expression regulation. As a fertilized egg develops into a human baby, signals received cause steady changes in gene expression patterns. Epigenetic tags physically record the cell's experiences on the DNA, and stabilize gene expression. Each signal activates some genes, and inactivates others, as the cell develops toward its final fate. Early in development, most signals come from within cells or from neighboring cells. Different experiences cause the epigenetic profiles of each cell type to grow increasingly different over time. Eventually, hundreds of cell types form, each with a distinct identity and specialized function. Specifc genes are turned on and off at certain time intervals, and any disruption of this finely-tuned DNA methylation regulation may persistently alter gene expression. The fetal epigenome is most susceptible during this developmental period to epigenetic modifiers in the maternal environment. An error during such a crucial time might lead to an abnormal phenotypic outcome in the offspring.

Assisted reproductive technologies (ARTs) like IntraCytoplasmic Sperm Injection (ICSI) and *in vitro* feritlization (IVF), which are used in case of male or female sub-fertility, cause epigenetic changes in offspring such that there is differential allelic expression in the early embryo (Kohda and Ishino, 2013). While comparing the two ARTs, ICSI, and IVF, both result in epigenetic errors owing to aberrant DNA methylation, but neither one has an increased effect as compared to the other (Santos et al., 2010). It has been shown that the use of ICSI results in hypermethylation of the Small Nuclear RiboNucleoprotein Polypeptide N gene (SNRNP) indicating likely mRNA splicing errors, which might result in such offspring (Whitelaw et al., 2014) (**Figure 6**).

Studies have also shown that ART can increase the risk of adverse pregnancy outcomes and imprinting errors resulting in incorrectly silenced genes (Bowdin et al., 2007; Reddy et al., 2007). As already mentioned in Section Endogenous and Exogenous Epigenetic Regulation of Genes, vitrification, which is also used in cryopreservation of embryos, leads to decreased methylation of OCT4 as well as other genes (Milroy et al., 2011; Zhao et al., 2012) further suggesting that ART is capable of altering the epigenetic profile, which might cause serious complications in future progeny.

#### Maternal influences

Maternal health can predict childhood development, health outcome and disease consequences. More specifically, fetal programming describes how the *in utero* environment impacts molecular development in the fetus via epigenetic remodeling (Barker and Clark, 1997; Schuz, 2010). Specific examples include the observation that reprogramming of the developing zygote involves conversion of 5-methylcytosine to 5-hydroxymethylcytosine (Wossidlo et al., 2011).

***Maternal diet.*** A mother's diet and stress influence the fetus *in utero* and can cause epigenetic changes as well. A study based on maternal folic acid supplementation showed that the offspring of mice fed with folic acid had a distinct global DNA methylation pattern compared to that of the offspring of mice which received a low folic acid dosage, involving genes associated with autism spectrum disorder (ASD) pathogenesis (Barua et al., 2014). The data supports the studies on the complexity of epigenetic regulation of genes GAD1 and RELN in ASD (Zhubi et al., 2014).

Also, experiments wherein maternal diet modification was the only variable were able to improve health outcome and increase longevity in offspring without changing DNA sequence. For example pregnant agouti mice, which are a strain predisposed to severe obesity, when fed diets supplemented with genistein, give birth to totally normal pups (Dolinoy et al., 2006).

Epigenetic effects of periconceptional diet on the DNA methylation status of rural Gambians shows that altered nutritional status of mothers during seasonal (weather) changes results in epigenetic variation in the three germ layers of offspring born during different seasons, including differential birth weight, and these changes persist through adulthood (Waterland and Jirtle, 2003).

***Maternal smoking.*** Other maternal effects include cigarette smoking, which during pregnancy can cause altered DNA methylation and micro RNA expression (Knopik et al., 2012).

***Maternal mental health and social environment.*** Maternal psychological health also exerts a powerful influence over the epigenetic outcome in offspring. In rats, prenatal stress during late gestation has been shown to modify epigenetic signatures that are linked to neurological disease during the critical period of fetal brain development (Zucchi et al., 2013). Also, domestic violence triggers stress in pregnant women that results in epigenetic changes in the DNA of the cortisol receptor in offspring observed during adolescence (Radtke et al., 2011).

#### Paternal influences

Environmentally induced epigenetic variation is also driven by paternal factors, and they are as important as their maternal counterparts in influencing epigenetic outcome in offspring (Carone et al., 2010; Hughes, 2014). During embryogenesis and fetal growth the insulin–like growth factor 2 (IGF2) gene is regulated by DNA methylation. Imprint marks are erased in primordial germ cells early in the process and new methylation patterns are created. Only paternal IGF2 is transcribed in normal tissues (Dechiara et al., 1991). New evidence indicates that paternal obesity is associated with a decrease in DNA methylation at *IGF2* (Soubry et al., 2013).

Also, DNA methylation in sperm can be influenced by paternal alcohol consumption, and paternal exposure to toxic chemicals such as vinclozolin and chromium chloride likewise alters the germ line epigenome (Cheng et al., 2004; Anway et al., 2005; Ouko et al., 2009; Stouder and Paoloni-Giacobino, 2010). Also, folate deficiency in male mice affects sperm function involving differential DNA methylation compared to control and the male offspring of such folate deficient mice show an altered gene expression compared to offspring born to control mice (Lambrot et al., 2013).

#### Developmental/in utero influences

Imprinted genes have trans-generationally stable DNA methylation patterns oblivious to the normal resetting that occurs early in normal development. These imprinted epigenetic marks are passed from parent to progeny on gametes, evading the normal epigenetic purging process that occurs during gamete formation, as described above. If a particular epigenetic profile is to pass to the next generation, the epigenetic tags associated with it must avoid erasure during reprogramming. Typically, a small minority of genes possesses epigenetic tags that survive the reprogramming process and pass unchanged from one generation to the next.

One such example is the neurological epigenetic disease called Angelman syndrome, that results from epigenetic silencing of the paternal UBE3 allele combined with a mutation or deletion in the maternal UBE3 allele, resulting in loss of E3 ubiquitin ligase, leading to multiple neuronal defects like epilepsy, ataxia, etc. (Rudenko and Tsai, 2014). Such epigenetic alterations based on comparison with a normal epigenetic profile can be considered useful biological markers for investigating neuropsychiatric symptoms of mental disorders with the help of new technologies like MeD-Chip sequencing (Fass et al., 2014).

Imprinted genes possess molecular memory of their germ line, associated with a variety of allelic DNA methylation patterns affecting genotype. But imprinted genes are still subject to epigenetic reprogramming following environmental exposures. Exposure to certain dioxin compounds induces DNA methylation in imprinted genes (Surani, 2001). Similarly, certain mammals respond to a hormonally triggered type of diabetes during pregnancy, known as gestational diabetes. Maternal gestational diabetes causes the developing fetus to be exposed to high levels of glucose. The high glucose in turn triggers epigenetic changes in the progeny DNA, often resulting in gestational diabetes in the next generation. Studies in rats with the proximal promoter region of *Pdx1*, a duodenal and pancreatic specific homeobox transcription factor reveal that onset of diabetes was associated with permanent silencing of the locus (Park et al., 2008).

In females, random X-chromosome inactivation is initiated during gastrulation in the epiblast through the X-inactive specific transcript (Xist) gene, which encodes a long non-coding RNA that silences the X-chromosome transcribing it (Reik, 2007).

Thus, as a fertilized egg develops into an embryo to fetus to a baby, numerous signals over the course of development cause incremental changes in expression of the genetic profile (**Figure 6**). Even in differentiated cells, signals will fine-tune cellular function by altering gene expression. Ultimately the flexibility inherent in the epigenome allows adjustments in response to the changing environment and the ability for future generations to successfully evolve and learn from earlier experience (Barlow and Bartolomei, 2014).

### Perinatal effects

Epigenetic influences continue to shape an individual after birth. Even at birth, the type of delivery seems to have an effect on the offspring being born. For example, offspring born from ceasarian section have shown to have global hypermethylation in leucocytes as compared to those born vaginally (Schlinzig et al., 2009).

#### Infancy and childhood

After birth and as life continues, a wider variety of environmental factors begin to play a role. As in early development, signals from within the body continue to be important for many processes, including physical growth and learning, but gradually more and more external environmental and social influences begin to take effect. For example, groundbreaking studies on the ability of behavior to modify the epigenome were conducted and demonstrated the important interplay between social and physical processes (Weaver et al., 2004). Early life positive and negative experiences like maternal care, stress adaptation, and early life adversities contribute to a biological memory, and epigenetic modifications of DNA are responsible for imprinting such influences in to the neuronal circuits of the developing brain which can have life-long impacts (Hoffmann and Spengler, 2014).

#### Maternal care and interaction

During infancy and childhood maternal care and social environment shape a child's psychology (Fagiolini et al., 2009). Maternal bonding has a profound effect on the physical and psychological welfare of children. Epigenetic mechanisms interact with and impact the hypothalamic-pituitary-adrenal axis of the stress response in the brain. For example, in mice, absence of maternal grooming epigenetically modifies DNA methylation, which alters glucocorticoid receptor expression. This leads to increased cortisol production by the adrenal glands and increases the stress response in pups (Plotsky and Meaney, 1993).

Also, murine maternal care like licking and grooming (LG) and arched back nursing (ABN) resulted in increased levels of hippocampal N-methyl D-aspartate receptor (NMDAR) and brain-derived neurotrophic factor (BDNF) in infant rats leading to increased neural synaptogenesis and enhanced spatial learning in adulthood (Meaney, 2001). Also pups experiencing frequent licking and grooming by their mother exhibit decrease in stress and anxiety through adulthood as a result of epigenetic changes at cortisol receptors (Weaver et al., 2004). Maternal care in rats has demonstrated decreased methylation of the offspring *Grm1* gene encoding metabotropic glutamate receptor (mGluR1) denoting epigenetic control of genes involved in glutamatergic synaptic signaling thereby influencing hippocampal function and cognitive performance (Bagot et al., 2012).

Licking by mother rats of their pups activates the NRC31 gene in the hippocampus of the brain and as a result protects the pups against stress (Oberlander et al., 2008; Bromer et al., 2013) (Figure 4). This epigenetically induced modification of DNA observed in rats is reversible, with remethylation of glucocorticoid receptors occurring in response to methionine injection (Waris and Ahsan, 2006). Presumably, therefore, it may be possible to prevent or reverse the onset of similar damaging epigenetic modifications in human children through behavioral therapy such as hugging, cuddling and other nurturing and stress-alleviating activities.

On the other hand, increased levels of corticotropin- release factor (CRF) in pups as a result of maternal separation was shown to be associated with serious mood disorders in adulthood (Meaney, 2001) There is similar evidence that suicide victims with a history of domestic violence and childhood abuse showed increased DNA methylation of the glucocorticoid receptor (NR3C1) gene leading to increased HPA (hypothalmis-pitutary-adrenal axis) activity resulting in elevated stress levels, also suggesting that suicide has a developmental origin (McGowan et al., 2009) (**Figure 6**).

Poverty and neglect have direct negative impacts upon future development (McGowan et al., 2009). The quality of family life including maternal care continues to influence the physiology and psychology of the child such that persistent neglect, emotional or sexual abuse hamper growth and intellectual development and increase risk of disorders like obesity during adulthood (Meaney, 2001).

More positively, epigenetic analysis of the serotonin transporter gene can be used for screening soldiers and identifying those at a greater risk for Post-traumatic stress disorder (PTSD) based on childhood trauma and thus specific epigenetic signatures can help in improvement of training regimens of such soldiers at a higher risk in order to avoid PSTD (Miller, 2011).

### Adolescence

The transition from childhood to adolescence is accompanied by temperamental and behavioral changes including emergence of sexual behavior which is driven by underlying hormonal changes that can also be influenced by environmental factors (Laviola et al., 2003). Puberty is a primary event of adolescence and is itself a major development event of human life. The HPG (hypothalamic-pitutary-gonadal) axis, which is dormant during childhood, is now activated (Seminara et al., 2003) which results in a sustained increase in gonadotropin-releasing hormone (GnRH) (Ojeda et al., 2010).

Puberty involves the maturation of certain regions of the pre-frontal cortex in the brain, and it has been suggested that environmental influences like stress can trigger neuropsychiatric diseases via epigenetic mechanisms during such vulnerable plastic development (Morrison et al., 2014). Puberty in females involves maturation of specific brain regions along with the increased expression of genes like KISS-1 (kisspeptin), NKB (neurokinnin B), GPR54, TAC3 along with decreased expression of two genes Eed and Cbx7 (polycomb group proteins) leading to the beginning of estrous cycle (Lomniczi et al., 2013; Morrison et al., 2014).

**Figure 5:**
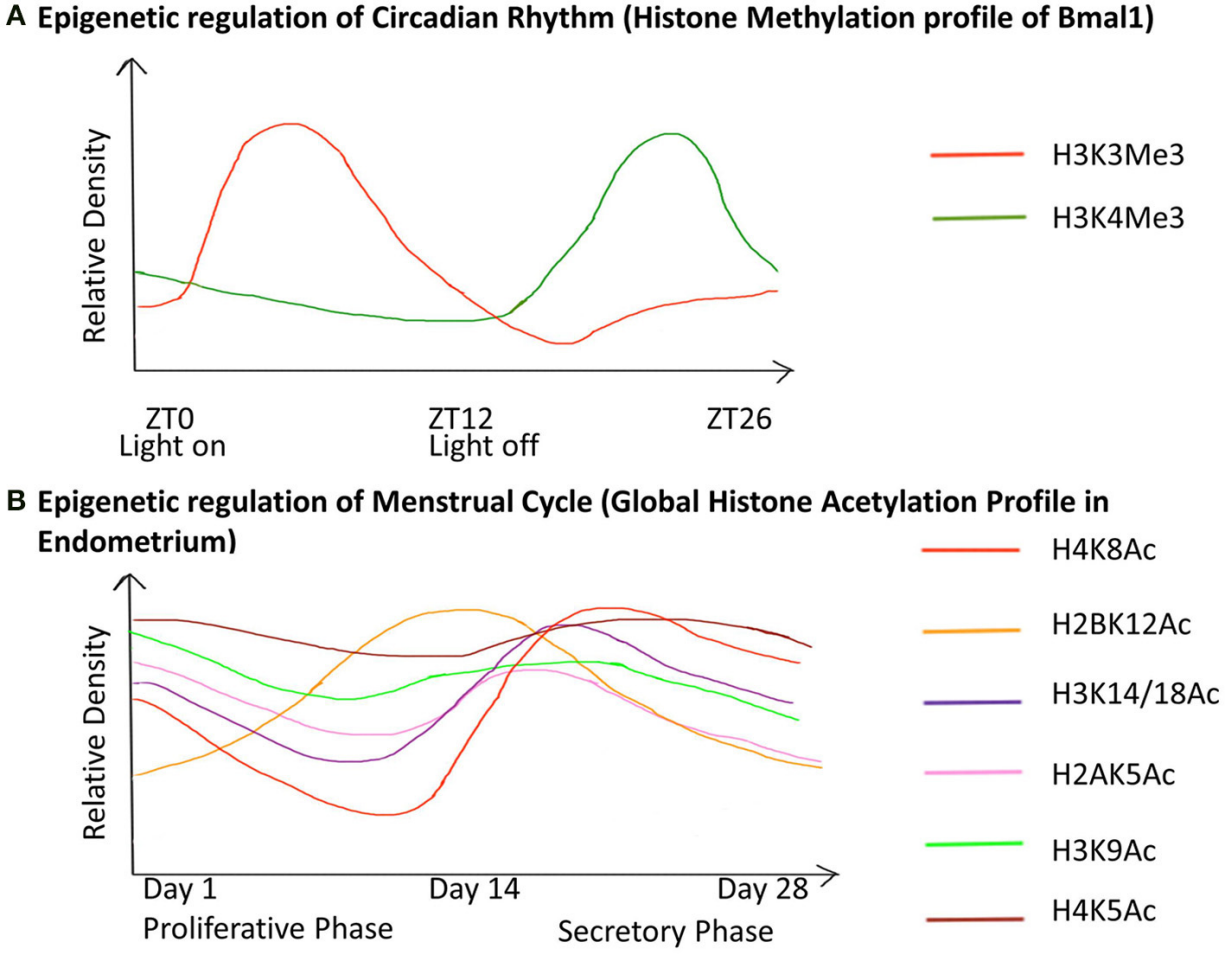
**Chronology of epigenetic regulation during the circadian rhythm and menstrual cycle**. The regulation of biological cycles like the Circadian rhythm and Menstrual cycle in females has been shown to work through epigenetic regulatory mechanisms. (The figure is relative and not accurate to scale). **(A)** The gene Bmal1 is the master regulator of the circadian rhythm. The graph represents the relative density of the two active histone marks H3K3me and H3K4me on Bmal1 through a 26-hour period. ZT is Zeitgeber Time where ZT0 corresponds to light on and ZT12 to light off. The peaks represent the maxima of the two-histone marks with respect to ZT, similarly, the dips represent the minima. This shows differential expression of the gene with respect to time, which has an epigenetic regulatory basis. **(B)** The graph represents the global acetylation profile from endometrial tissue through the 28-day menstrual cycle. The peaks represent the maxima and the dips represent the minima of the histone marks with respect to time. Global changes in the histone acetylation profile show that these marks contribute to the induction of different genes during different stages of the cycle. Hence they are responsible for the variable morphology of the endometrium through the 28-day period. These epigenetic changes are thus the basis for changes in the endometrial morphology like regeneration (1–7 days), proliferation (7–14 days), ovulation (13–17 days), differentiation (15–22 days), degradation (22–28 days) during the proliferative phase and secretory phase.

Puberty in females also leads to menarche i.e., the beginning of the menstrual cycle in females. The menstrual cycle is an orchestrated event that operates through a 28 day period during which the endometrium undergoes cyclic morphological transformations of growth, differentiation and regression (Munro et al., 2010). We have illustrated the epigenetic fluctuations (global histone acetylation) governing the gene expression in the endometrium through this cycle (Figure 5).

### Adulthood

Early life experiences modify the neurobiology of development and such influences continue to affect behavioral patterns and psychological outcomes in adulthood (Roth, 2012). In addition, various external epigenetic factors modulate the biology of an individual at a physical and emotional level. Some of the most important exogenous factors influencing human health are described hereafter. We have described these influences in the “Adulthood” section for the sake of narrative simplicity, although they actually apply across the entire lifespan.

#### Diet

Studies in genomic imprinting have revealed how DNA methylation patterns are influenced by diet, and how epigenomic sensitivity to environmental cues and specifically diet can be used to influence disease susceptibility (Waterland and Jirtle, 2003; Carone et al., 2010; Jennings and Willis, 2014).

Nutrients extracted from the diet enter metabolic pathways and are transformed into useful molecules. These nutrients are known to have epigenetic targets in cells such that they can be used to modify the epigenome in order to correct abnormally activated or silenced genes and can be combined into an “epigenetic diet” useful as a therapeutic or chemopreventive measure (Hardy and Tollefsbol, 2011). During this transitory phase methyl groups are formed from key nutrients including folic acid, B vitamins and s-adenosyl methionine (SAMe), and these methyl groups, as described earlier, comprise important epigenetic marks for gene silencing. Diets high in such methyl rich nutrients may significantly alter gene expression and offer protective health benefits. Deficiencies in folate and methionine, both of which are involved in cellular processes that supply methyl groups needed for DNA methylation, can change the expression (imprinting) of growth factor genes such as (IGF1).

***Folic acid.*** As explained earlier, DNA methylation can be used to distinguish a healthy CpG methylation profile from its inverted counterpart in tumor cells. Certain DNA repair genes including MGMT and MLH1 are prone to transcriptional silencing by promoter hypermethylation (Jones and Baylin, 2002; Esteller, 2007). Variables including diet are capable of influencing epigenetic programing by altering DNA methylation and interfering with gene expression. Such potentially heritable modifications can be regulated by diet-responsive methylation. Specifically, deficient levels of folic acid lead to epigenetic alterations by inhibiting remethylation of s-adenosyl homocysteine (SAH) and s-adenosylmethionine (SAM) which results in demethylation and chromosome instability (Dulthie, 2011). Thus, not only can dietary folate bolster a healthy locus-specific and global DNA methylation program, but can also direct proper uracil incorporation, inhibit DNA breakage, and foster DNA repair via thymidine and purine biosynthesis (Ingrosso et al., 2003). Dietary folate present in a variety of green vegetables including broccoli, zucchini, brussels sprouts, green beans and spinach participates in maintaining a healthy DNA methylation profile and even reverses accrued damage (Bhusari et al., 2010; Jennings and Willis, 2014).

***Antioxidants and phytochemicals.*** Disruption in the balance between reactive oxygen species and antioxidants may result in harmful health effects caused by DNA damage due to the genotoxic effects of oxidative stress (Waris and Ahsan, 2006). Foods are known to alter epigenetic expression in rats on different diets. Chemopreventive agents that target the epigenome include micronutrients found in folate, retinoic acid, selenium compounds, polyphenols from green tea, apples, coffee, black raspberries, and other dietary sources. Similar compounds are present in foods containing genistein and soy isoflavones, curcumin, and resveratrol to name a few (Gerhauser, 2013). While certain food components epigenetically increase the levels of DNA repair enzymes such as MGMT and MLH1, others such as soy, isoflavones and bilberry anthocyanins actively decrease DNA damage (Davis et al., 2001; Djuric et al., 2001; Olaharski et al., 2005; Fang et al., 2007; Kikuno et al., 2008; Burdge et al., 2011; Kropat et al., 2013). Anthocyanin is an effective antioxidant for humans that is found in plants and are easily identified by its potent red or purple pigment. It is found in plants such as eggplant, plums, pomegranate, red onion, cranberries, blueberries, kidney beans and cherries which all possess anthocyanins. This flavonoid serves as a powerful antioxidant that contributes to scavenging of DNA-damaging free radicals. While the direct fate of anthocyanins *in vivo* following digestion may be less than 5% (the majority being rapidly excreted), the potent residual antioxidant property remains in blood following consumption of anthocyanin-rich foods due to metabolic breakdown of the flavonoids and resultant increase in uric acid levels (Williams et al., 2004; Lotito and Frei, 2006).

Another example is the polyphenol epigallocatechin-3-gallate (EGCG), which is contained in green tea and has been shown to retard carcinogenesis (Fang et al., 2003). The pathway is similar to that used by other foods such as genistein in soy and involves regulation of DNA methylation at key genes to elicit positive epigenetic outcomes. Others like sulfopropanes from cruciferous vegetables and green tea can be used to treat age-related diseases and cancer since they are capable of reverting an aberrant epigenetic profile (Tollefsbol, 2014). Other like sulfopropane form Owing to the numerous advantages that can be obtained through foods containing beneficial components and from probiotics, nutri- and microbial epigenetics is growing in importance because these bioactive molecules have been shown to be involved in regulating chromatin receptors and epigenome-related pathways (Shenderov and Midtvedt, 2014).

#### Social interaction and behavior

There is no sharp edge dividing the biological from the behavioral aspects of epigenetic research. In fact there is a great deal of overlap. However, many interesting social perspectives contribute to the role of behavior on epigenetic modifications, and as such warrant a separate discussion since behavior is more readily controlled than biology. When mice were subjected to odor-fear conditioning prior to conception, increased behavioral sensitivity was observed in offspring in subsequent two generations (Dias and Ressler, 2014). Epigenetic modifications complement the genome in that they do not change the DNA code directly, but influence it in such a way as to present it to the factors that read it and translate it into a final product. The ability to create, process and recall memory is reliant in part upon epigenetic modifications such as DNA remodeling (Lattal and Wood, 2013). Nucleosome remodeling where histone-dependent nucleosomes are repositioned to expose DNA for gene expression is a factor in both healthy memory formation and cognitive impairment (Malvaez et al., 2013; Vogel-Ciernia et al., 2013).

#### Exercise

Exercise is one way that an individual can modify their epigenome in order to preserve and prolong life. Exercise has been shown to induce positive changes in DNA methylation within adipose tissue and regulate metabolism in both healthy and diseased individuals (Ronn et al., 2013). Increased DNA methylation of genes Hdac4 and Ncor2 has also shown to increase lipogenesis following exercise (Ronn et al., 2013). Exercise also leads to beneficial changes in DNA methylation patterns in skeletal muscle (Nitert et al., 2012). Not only is obesity an indicator for diseases such as type 2 diabetes and cardiovascular disease, but also puts additional stress on the system which can itself negatively impact health (Ronti et al., 2006). Acute exercise is associated with DNA hypomethylation of the entire genome in skeletal muscle cells of sedentary individuals and high intensity exercise tends to cause reduction in promoter methylation of certain genes (Ntanasis-Stathopoulos et al., 2013). Exercise is also known to positively influence the expression patterns of miRNAs in leukocyte cells (Radom-Aizik et al., 2010). The health benefits of physical exercise, especially on a long term and strenuous basis, has a positive effect on epigenetic mechanisms and ultimately may reduce incidence and severity of disease (Sanchis-Gomar et al., 2012).

#### Pharmaceutical drugs

Pharmacoepigenetics is the study of inter-individual variations in epigenetic modifications as a result of prescribed pharmaceutical drug use. One working hypothesis is that exposure to therapeutic drugs may cause persistent epigenetic changes, possibly manifesting as permanent adverse side-effects (Csoka and Szyf, 2009). There is a long list of drugs for which clinical or experimental evidence exists documenting either direct or indirect epigenetic effects (as described above). Direct effects are caused by drugs that interfere directly with the normal controls of DNA and/or histone methylation, resulting in aberrant gene expression. Indirect effects cause epigenetic changes by interaction with a cell surface receptor, enzyme, or other protein, which thereby alters expression of said receptors, and subsequently alters expression of transcription factors, which in turn change epigenetic regulation. Several epigenomic screening protocols are in place to identify drugs whose epigenetic power has therapeutic benefit and to isolate other drugs whose negative epigenetic impact outweighs potential benefit.

#### Drugs of abuse

Many drugs are used to enhance or alter the perception of reality and reward pleasure centers in the brain, but oftentimes these substances increase the risk of acute and/or chronic disease. Generally speaking, recreational drugs like cocaine but also including opiates, amphetamines, alcohol and nicotine modify the epigenome by altering methylation patterns in areas such as the nucleus accumbens of the brain, the major pleasure reward center (Renthal and Nestler, 2008; Maze and Nestler, 2011; Doehring et al., 2013). Recreational drugs such as cocaine induce epigenetic changes in many ways, such as increasing histone acetylation on c-fos and fosb gene promoters (Kumar et al., 2005). In terms of histone methylation, the epigenetic mark H3K9 been associated with chronic cocaine use, as well as with the process of cocaine addiction. Cocaine-induced plasticity is associated with the reduction in H3K9 methylation marks due to the repression of HMT G9a in the nucleus accumbens region of the brain (Maze et al., 2010). Similarly, it has been demonstrated that pretreatment with histone deacetylase (HDAC) inhibitors helped in curbing cocaine addiction in animal models (Romieu et al., 2011).

Smoking causes epigenetic changes such as DNA methylation changes, which alter gene expression. For example cigarette smoke induces demethylation of metastatic genes in lung cancer cells by downregulating DNMT3B (Liu et al., 2007). DNA methylation is a type of epigenetic change that can result in tumor suppressor genes being inactivated, and methylation of the tumor suppressor gene p16 has frequently been associated with the development of cancer. When p16 is methylated, this gene's tumor suppressing function undergoes inactivation. This is why smoking is a major trigger for carcinogenesis. Of particular interest is the finding that not only maternal smoking but also grandmaternal smoking is linked to pediatric disease as a result of epigenetic changes to DNA and histones (Rehan et al., 2012; Leslie, 2013) (See below for description of trans-generational influences).

***Alcohol.*** The epigenetic effects of alcohol on hepatic and neuronal tissue are well documented (Shukla et al., 2008). Ethanol is known to cause site-selective methylation, acetylation and phosphorylation of histones and DNA hypomethylation due to reduction of tissue SAM (Shukla et al., 2008; Zakhari, 2013). One carbon metabolism (OCM) is a major methyl group donor, contributing toward DNA methylation and alcohol's interference with OCM leads to reduced availability of methyl groups and hence aberrant DNA methylation and gene expression in alcohol consumers (Kruman and Fowler, 2014). A study with young mice showed that chronic alcohol exposure reduced global DNA hydroxymethylation in the liver as compared to control (Tammen et al., 2014). Alcohol induced changes at the gastrointestinal- hepatic level like steatosis, carcinogenesis, endotexemia are also a result of epigenetic alterations (Shukla and Lim, 2013).

Alcohol induced neuro-adaptations like tolerance and dependence are a result of epigenetic modulation at the neurobiological level (Finegersh and Homanics, 2014a). Acute ethanol exposure in mice leads to decreased expression of GAD1, HDAC2, HDAC11 associated with decreased histone acetylation at GAD1, HDAC2 promoters and increased expression of MT1, MT2, EGR1 associated with increased levels of H3K4me3 at MT2 promoter and decreased level of H3K27me3 at the MT1 promoter in the cerebral cortex (Finegersh and Homanics, 2014a).

Paternal alcohol exposure, prior to mating, has shown to induce increased sensitivity to anxiolytic and motor effects of alcohol, reduced alcohol preference and consumption exclusively in male offspring in mice (Finegersh and Homanics, 2014b). An increased expression of BDNF in the ventral tegmental area of such male offspring was also observed along with DNA hypomethyaltion of the BDNF promoter in the sire's germ cells and male and female offspring (Finegersh and Homanics, 2014b). Such effects are epigenetically transmitted through the male lineage and are capable of being a trans-generational influence. (See below for description of trans-generational influences.) Recent efforts toward elucidation of exact interactions between alcohol and the epigenome will help in developing treatments for alcohol-related disorders including fetal alcohol spectrum disorders, alcohol addiction and organ damage (Shukla and Zakhari, 2013).

In recent years the interest in the identification of epigenetic marks associated with alcohol dependence has been growing (Starkman et al., 2012). A major obstacle in this research is the limited number of candidate structural genes that might be participating in these pathways. However, DNA methylation in relation to alcohol use disorders has lately become a burning topic of research; a significant association between loss of control over drinking behavior and DNA methylation at multiple CpG sites has been observed, specifically methylation at CpG sites near the ALDH1A2 gene was found to be associated with rate of intoxication and inclination toward alcohol (Harlaar et al., 2014).

#### Alternative medicine

Alternative medicine (AM) approaches like ayurverda, homeopathy, yoga, taichi, reiki, acupuncture, body massages, naturopathy, and hypnotherapy offer a promising approach toward improving human health and lifestyle by mediating beneficial environment-epigenome interactions (Figure 1). AM includes a variety of health practices, knowledge, beliefs and moreover therapeutic as well as spiritual techniques, which can be used to prevent or treat health conditions (Report, 2001). One of the well-known forms of AM is Ayurveda, which has emphasized personalized health care for over 5000 years. Ayurvedic science advocates the use of herbs and spices in addition to foods for their medicinal properties, and their epigenetic effects have been identified. For example, tulsi and ginger regulate histone H3 acetylation and others spices such as turmeric and cinnamon possess similar effects (Shim et al., 2011). Acupuncture, another form of AM, has been noted for its beneficial effects on cardiac health. Acupuncture therapy has been used for prevention of myocardial infarction (MI), strengthening of cardiovascular function and angiogenesis, and protection from further injury and apoptosis to myocardial cells after an MI (He et al., 2014). This suggests how alternative medicine may operate through epigenetic regulation of regeneration, or cellular apoptosis in case of injury or degenerative diseases.

#### Environmental chemicals

Encounters with pesticides, toxins and synthetic compounds can methylate genes in adults and also promulgate diseases decades later in offspring following *in utero* exposures.

Heavy metals including arsenic, nickel and cadmium are widespread environmental contaminants capable of disrupting DNA methylation and histone acetylation and as a result have been associated with a number of diseases including cancer, neurological disorders and autoimmune diseases. Putative mechanisms may involve the fact that metals act as catalysts in the oxidative deterioration of biological macromolecules to produce free radicals and induce epigenetic changes (Leonard et al., 2004; Babar et al., 2008; Monks et al., 2006).

Arsenic undergoes metabolic detoxification via methylation by *S*-adenosyl methionine (SAM), a universal methyl donor for methyltransferases (including DNMTs). Exposure to arsenic both decreases the activity of DNMTs and down regulates DNMT gene expression (Reichard et al., 2007). Arsenic exposure induces hypermethylation of tumor suppressor genes (Jensen et al., 2008), dysregulation of histone acetylation (Hou et al., 2012), and promotion of micro RNA expression (Marsit et al., 2006). Nickel is capable of compacting chromatin and methylating the DNA of tumor suppressor genes (Lee et al., 1995). Exposure to nickel affects histone acetylation causing gene silencing and ultimately cell transformation (Broday et al., 2000; Zhang and Zhu, 2012). Exposure to cadmium, mercury, lead, and chromium cause similar epigenetic modifications (Takahashi et al., 2005; Huang et al., 2008; Sun et al., 2009; Wright et al., 2010; Arai et al., 2011).

Bisphenol A (BPA) and phthalates are plastics that leach into the environment. Not only are they estrogenic and known endocrine disruptors, but these chemicals and their metabolites also induce epigenetic modification in animal models (Singh and Li, 2012). When ingested by humans, diethylhexyl phthalate (DEHP) is converted by intestinal lipases to mono-(2-ethylhexyl) phthalate (MEHP), which is then preferentially absorbed (Morgan et al., 1999). Specifically, BPA disrupts DNA methylation by decreasing CpG methylation in the mouse agouti gene. This effect is reversible through dietary supplementation with a source of methyl donor groups such as folic acid and the phytoestrogen genistein (found in fava beans, soy beans, and coffee) (Dolinoy et al., 2006).

#### Seasonal and diurnal changes

The operation of the circadian rhythm in humans is a result of temporal expression of clock controlled genes (CCGs) (Sahar and Sassone-Corsi, 2012). Chromatin remodeling is the basis for differential expression of such CCGs through a 24-hour cycle, for example the epigenetic regulation Bmal1 (Figure 5). The drastic changes in lifestyle through the last century and higher incidence of modern day diseases like obesity, Type 2 diabetes and a spectrum of mental disorders has been linked to disruption of the internal circadian clock, a mechanism of homeostatic regulation of physiological processes and metabolic functions, and this has been attributed to exposure to epigenetic factors (Orozco-Solis and Sassone-Corsi, 2014).

It is known that, unlike humans, the changes in season affect the reproductive behavior of seasonal breeding vertebrates as a physiological response to an altered photoperiod exposure. A study with Siberian hamsters showed that nocturnal pineal melatonin (MEL) protein is involved in DNA methylation changes of the promoter of dio3 gene, which is responsible for photoperiodic time measurement, and undergoes reversible epigenetic alterations for physiological orientation in time (Stevenson and Prendergast, 2013).

#### Menopause

**Figure 6:**
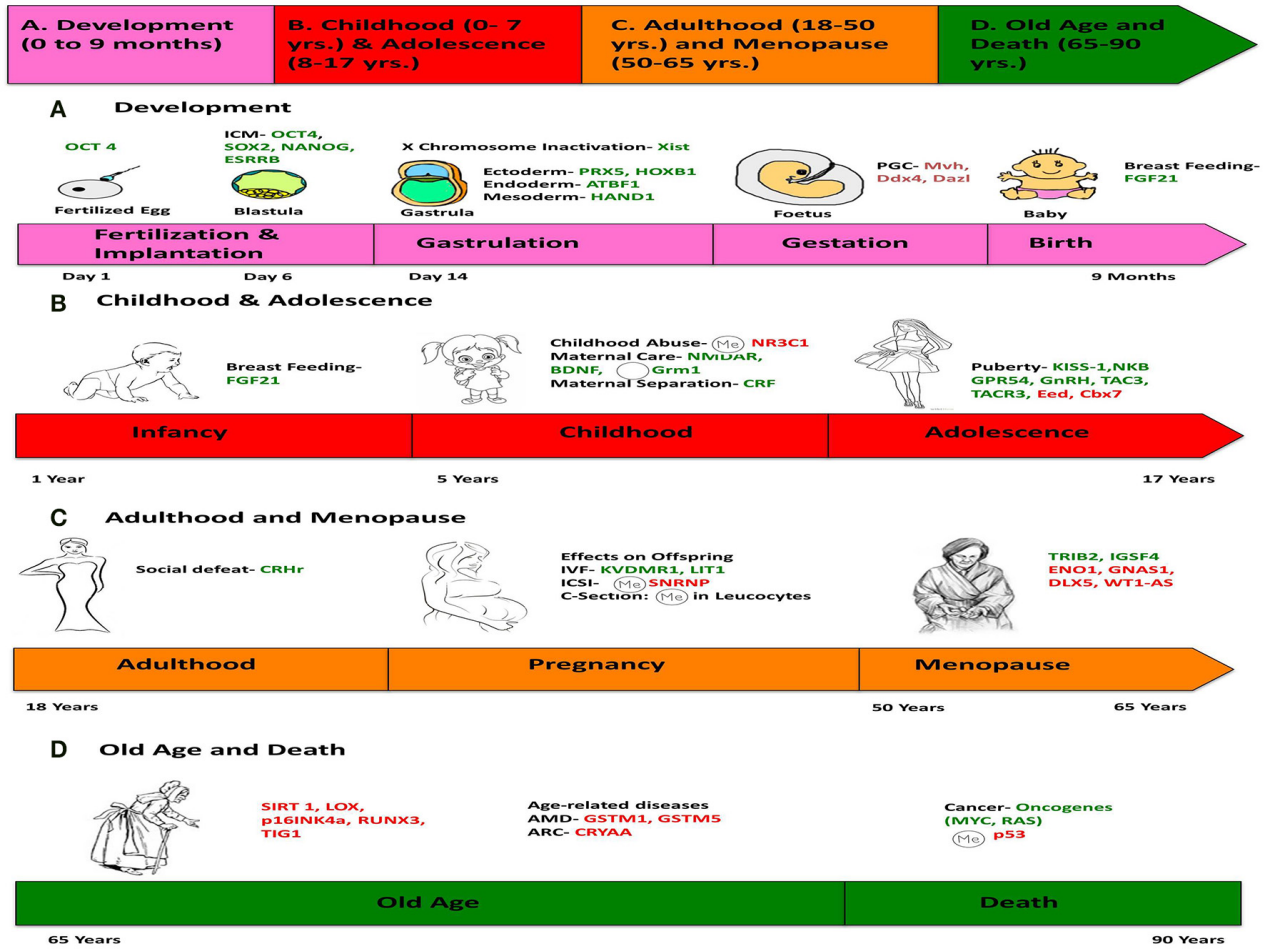
**Chronology of epigenetics during the life of a human female**. This figure is a summary of the most important events in the lifetime of a human female, which contribute toward her physical and mental development, and the genes responsible for such changes are listed. (We chose a female because the lifetime of a female has more descriptive events compared to a male). The life of a female can be divided into four stages: **(A)** Development (0–9 months), **(B)** Childhood (0–7 years) and Adolescence (7–17 years), **(C)** Adulthood (18–50 years) and Menopause (50–65 years), **(D)** Old Age and Death (65–90 years). Genes in green represent those, which are expressed/induced, and the ones in red represent the genes repressed/silenced during a particular stage of life, and contributing to a specific phenotype. The symbol 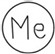 denotes hypermethylation and 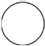 denotes hypomethylation, usually of the promoter of the gene. For example, puberty is initiated when genes like KISS-1 (shown in green) is induced. Aging is associated with silencing or down-regulation of SIRT1 gene (represented in red). [PCG, primordial germ cells; FGF21, Fibroblast growth factor 21; ARC, Age-related cataract; CRYAA, chaperone-like activity of αA-crystalline; AMD, Age-related macular degeneration; GSTM, Glutathione S-transferase isoform mu1 (GSTM1) and mu5 (GSTM5)].

Menopause is an event in a female's life that results in termination of her reproductive capability and is a gradual process accompanied by the deregulation of hormones (Ubeda et al., 2014). Many genes are known to be epigenetically regulated during this process, like those implicated in DNA repair (EXO1, HELQ, UIMC1, FAM175A, FANCI, TLK1, POLG, and PRIM1) and immune function (IL11, NLRP11, and PRRC2A) (Stolk et al., 2012). ENO1 is downregulated after menopause whereas TRIB2 and IGSF4 are up regulated (Kosa et al., 2009) (Figure 6).

It is known that the DNA methylation status of Polycomb group target (PCGT) genes in endometrial tissue can be used as a biomarker to predict the risk for cancer development in premenopausal women (Widschwendter et al., 2009). One of the members of the PCGT group, namely HOXA10, is highly expressed during implantation and is important for the process and its expression is reduced in patients with endometriosis (Bagot et al., 2000; Wu et al., 2005). It was found that the global DNA methylation of eutopic endometrium in patients with endometriosis was higher as compared to ectopic endometrium of patients with endometriosis and a control group (Andersson et al., 2014). Overall, aberrant DNA methylation leading to aberrant gene expression plays an important role in disease states of the endometrium and can cause additional changes to the process of menopause in females.

## Aging and age-related disease

Aging is a multifactorial process that results in a progressive loss of regenerative capacity through a decline in cell-proliferation and tissue functionality (Campisi, 2013). It induces global and complex changes in the human DNA methylation landscape (Xu and Taylor, 2014). Additional epigenetic changes have been described which provide clues to understanding the aging process. For example, senescent cells are characterized by formation of a specialized heterochromatin called senescence-associated heterochromatin foci that exhibit noticeable histone modifications (Narita et al., 2003). These foci contribute to senescence-associated proliferation arrest that may silence the expression of proliferation-promoting genes (Sedivy et al., 2008). Shortened telomeres, a hallmark of aging, may have reduced heterochromatin (Benetti et al., 2007).

### Genome-wide DNA methylation changes

Generally, during the aging process, global hypomethylation of DNA occurs in a repetitive sequence pattern that may promote genomic instability (Heyn et al., 2012). Not only is aging correlated with hypomethylation of proto-oncogenes, but also with hypermethylation of tumor suppressor genes, potentially leading to increased risk of cancer and other diseases (Coppede, 2014).

Some methylation changes are so predictable that age-related DNA methylation patterns have been used to predict an individual's actual chronological age, such that DNA methylation can be used as a measure of age or age acceleration, as so-called “aging clock” (Horvath, 2013).

Epigenetic changes in disease are not always focal, but can be global and encompass large chromosomal regions. For example the aberrant expression of micro RNAs has been linked to various age-related diseases such as Alzheimer's disease, cardiac disease and many cancers including leukemia and lymphoma (Natarajan et al., 1992; Fabbri et al., 2009; Cheng and Zhang, 2010; Montgomery and Van Rooij, 2010; Provost, 2010), as described below.

### Alzheimer's disease

Alzheimer's disease is a neurodegenerative disorder characterized by β-Amyloid, inflammatory, oxidative and vascular damage. DNA hypomethylation of the entorhinal region of the cerebral cortex, plus hypermethylation and consequent overexpression of the hTERT gene have been reported in Alzheimer's research (Silva et al., 2008; Mastroeni et al., 2010). Animal studies suggest that overexpression of histone deacetylase 2 (HDAC 2) may decrease synaptic plasticity and impair memory formation (Guan et al., 2009; Urdinguio et al., 2009). Also, DNA methylation deficiency can accelerate age-related diseases and it has been shown that Dnmt1 haploinsufficiency can impair learning and memory function causing cognitive disorders with age (Liu et al., 2011).

### Arthritis

The epigenetic basis of osteoarthritis (OA) is seen in the fact that OA chondrocytes express genes involved in degradation of cartilage as a result of their hypomethylated promoters under the influence of cytokines like IL-1β and TNF-α, as opposed to normal chondrocytes (Haseeb et al., 2014).

### Cardiovascular disease

Heart disease is often attributed to genetic predisposition, but epigenetic marks that vary between cell types and respond to endogenous and exogenous stimuli likely share culpability (Ordovas and Smith, 2010). DNA methylation is critical for the development of atherosclerosis and cardiovascular disease; recently hypermethylation has been shown in differentially methylated genomic regions of patients suffering with coronoary artery disease (CAD) (Sharma et al., 2014). DNA methyltransferases (*DNMT*s) exhibit hypomethylation in mice, an observation associated with increased expression of inflammatory mediators (Makar and Wilson, 2004). Also, DNA hypermethylation has been observed in the estrogen receptor genes ESR1 and ESR2 of vascular smooth muscle which contributes to atherosclerosis (Lund and Zaina, 2011).

Not only is DNA methylation a primary regulator of inflammation, but also controls leukocyte functions related to cardiovascular risk (Baccarelli et al., 2010). Removal of epigenetic signatures of oxidative and inflammatory genes has been proposed as a promising therapeutic option to prevent endothelial dysfunction and vascular complications in diabetic people (Paneni et al., 2013).

Histone modification has also been implicated in various aspects of cardiovascular disease such as angiogenesis and myocardial infarction (Webster et al., 2013). Histones are important for the normal regulation of the NOS3 gene. This gene codes for the protein eNOS that catalyzes formation of NO, a vasodilating factor in blood vessels that operates in the regulation of healthy cardiovascular tissue and can inhibit the ability of KDM3a protein to remove histone methyl groups (Hickok et al., 2013).

### Cancer

Cancer has long been considered a disease of genetic origin, but an historic link between cancer and epigenetics was identified when DNA of colorectal cancer patients was observed to be hypomethylated (Feinberg and Vogelstein, 1983). The normally hypermethylated and silent regions of the genome which contain repetitive sequences become demethylated. Conversely, hypermethylation of CpG islands in certain cancers is correlated with abnormal gene activity, such as deactivation of tumor suppressor genes (Esteller, 2002). Hypermethylation of DNA also damages repetitive sequences of DNA called mircosatellites (Oki et al., 1999); hypermethylation of the MLH1 promoter (a DNA repair gene) disfigures microsatellites, a phenomenon present in many cancers including colorectal and ovarian (Jones and Baylin, 2002). These observations of promoter hypermethylation (Coppede, 2014) and heterochromatinization (Rideout et al., 1994) are compelling evidence of the dramatic influence of epigenetics on tumorigenesis.

A major locus susceptible to transcriptional silencing as a result of promoter hypermethylation is the INK4 locus located on chromosome 9 (Kim and Sharpless, 2006). INK4 represents a family of cyclin-dependent kinase inhibitors. This gene encodes several proteins that are often targeted early during malignant progression, including p14, p15, and p16. Histone modification is another epigenetically important mechanism prevalent during carcinogenic transformation. Transcriptional control of p16 is interrupted when chromatin domains are lost and discrete histone structure is destroyed, in breast cancer cells for example (Witcher and Emerson, 2009).

Epigenetic changes differ from their genetic counterparts such as gene mutations in that epigenetic hypermethylation affects many genes within a single cancer cell (Coppede, 2014). For example, PIAS1, a transcriptional repressor, is involved in the progression of breast cancer and operates on an epigenetic level through gene silencing by recruting DNMTs (Liu et al., 2014). Still, many researchers view the link between cancer and epigenetics with optimism, since epigenetic modifications are potentially reversible. For example, it is thought that cells harboring gene mutations must be killed or removed to prevent uncontrolled propagation of the damaged code. But advances in epigenetic technology may soon allow repair of defective epigenetic modifications by a variety of therapeutics. For example the drug azacitidine, the first FDA-approved epigenomic drug, treats leukemia by reactivating tumor suppressor genes and similar drugs are now in development (Braiteh et al., 2008; Phillips, 2008).

## Trans-generational influences

Many transgenerational phenotypic inheritance profiles cannot be explained by the normal human mutation rate *of* 2.3 × 10^−8^ per nucleotide per generation (Arnheim and Calabrese, 2009). The reason for this discrepancy is that epigenetic changes most likely contribute to the majority of these transgenerational effects.

Transgenerational epigenetic inheritance was first observed in plants (Manning et al., 2006), however, it has also been reported in rodents and humans (Carone et al., 2010). Heritability of epigenetic expression is demonstrable where multiple generations are simultaneously exposed to the same environmental conditions that include diet, toxins, hormones, etc. (Curley et al., 2011; Vassoler et al., 2013). In such a model, the mother = first generation, the fetus = 2nd generation, the reproductive cells of fetus = 3rd generation, and the future offspring of fetus = 4th generation. In order to provide a convincing case for trans-generational stability, an epigenetic change must be observed in the 4th generation (Hughes, 2014). Recent studies have shown that maternal methyl-donor supplementation affects not only the mother, but is also inherited in the F2 generation through germline epigenetic modifications (Duhl et al., 1994; Wolff et al., 1998; Morgan et al., 1999; Cropley et al., 2006). Transgenerational epigenetic influence of longevity has also been shown in *C. elegans* (Greer et al., 2011).

## Epigenetics in translational and personalized medicine

For the past several decades genetics has been at the forefront in terms of understanding human disease. A recent addition to genetics has been epigenetics, which includes the role of the environment, both social and natural, including day-to-day habits, lifestyle and personal experiences on human health. Epigenetics establishes a scientific basis for how external factors and the environment can shape an individual both physically and mentally.

The knowledge that environment and lifestyle can alter health brings with it awareness that habits, social environment, diet and other factors shape health beyond our acquired genetic traits. Moreover, despite the risk presented by inherited genes and mutations, epigenetic factors play a decisive role in the actual development of disease. Research into epigenetics could lead to insights into how factors like diet and exercise can be customized to an individual in concordance with their naturally inherited genome in order to minimize the risk of developing a disease to which he/she is naturally predisposed.

Advances in epigenetic profiling technology such as genome-scale DNA methylation analysis points to the future possibilities of how epigenetic profiling can help in determining the risk of an individual with a particular type of genetic makeup developing a specific disease. Also the same epigenetic profile, along with knowledge of the genomic sequence, can help to determine which medications or alternative medicine approaches would be effective in preventing or curing a particular disease. Such approaches toward improving health and lifestyle and reducing the risk of developing an otherwise inevitable disease might be possible in the near future. It will require extensive knowledge of the biochemical and physiological mechanisms of epigenetics, and we are still on this challenging path, but we have good reasons to be optimistic.

## Conclusions

We have presented a comprehensive narrative of the role of both endogenous and exogenous epigenetic influences on the human lifespan.

The future of epigenetics holds tremendous promise for understanding the complexities involved in genetic regulation, cellular differentiation, aging and disease; and a more complete and comprehensive understanding of the mechanisms that underlie the formation and erasure of epigenetic marks could allow us to commandeer the process and possibly fine tune the human epigenome.

Ultimately, continued efforts to determine how and when epigenetic switches regulate gene function will elucidate the interplay between the genome, the epigenome, and the environment and facilitate the development and optimization of novel therapeutic tools. In terms of future application, full understanding of these mechanisms will ultimately revolutionize personalized medicine.

### Conflict of interest statement

The authors declare that the research was conducted in the absence of any commercial or financial relationships that could be construed as a potential conflict of interest.
